# Cross-Cultural Adaptation and Psychometric Evaluation of the Turkish Short Falls Efficacy Scale–International (Short FES-I) for Assessing Concerns About Falling in Community-Dwelling Older Adults

**DOI:** 10.3390/healthcare14213694

**Published:** 2026-11-01

**Authors:** Berna Bilgin Şahin, Hüseyin Elbi, Yusuf Adnan Güçlü, Sıdıka Ece Yokuş, Ali Hüseyin Doğan

**Affiliations:** 1 Department of Public Health, Faculty of Medicine, Manisa Celal Bayar University, 45030 Manisa, Turkey; 2 Department of Family Medicine, Faculty of Medicine, Manisa Celal Bayar University, 45030 Manisa, Turkey; huseyinelbimd@gmail.com; 3 Department of Family Medicine, Tepecik Training and Research Hospital, University of Health Sciences, 35180 Izmir, Turkey; ya_guclu@hotmail.com; 4 Bayrampasa Yildirim Family Health Center, 34045 İstanbul, Turkey; yokusece@gmail.com; 5 Ağrı Patnos District Health Directorate, 04500 Ağrı, Turkey; alihuseyindogan6@gmail.com

**Keywords:** Short FES-I, fear of falling, concerns about falling, older adults, validity, reliability

## Abstract

**Background/Objectives**: This study aimed to evaluate the psychometric properties of the Turkish version of the Short Falls Efficacy Scale–International (Short FES-I) in community-dwelling older adults. **Methods**: This cross-sectional methodological study included 200 adults aged ≥65 years recruited from Family Health Centers. Following translation and cross-cultural adaptation, internal consistency was assessed using Cronbach’s alpha, McDonald’s omega, and corrected item–total correlations. Structural validity was examined using confirmatory factor analysis (CFA) with diagonally weighted least squares estimation, including an initial single-factor model and a modified model incorporating two post-hoc correlated residuals. Construct validity was evaluated through hypothesis testing, including Spearman correlations and known-groups comparisons. Multiple linear regression was performed as a supplementary analysis of factors associated with Short FES-I scores. **Results**: The Turkish Short FES-I demonstrated high internal consistency (Cronbach’s α = 0.910; McDonald’s ω = 0.915), with corrected item–total correlations ranging from 0.608 to 0.839. The initially specified single-factor CFA model showed an elevated RMSEA (0.113). Following the introduction of two post-hoc correlated residuals between Items 4 and 6 and between Items 5 and 7, the modified model showed improved fit (CFI = 0.999, TLI = 0.998, SRMR = 0.045, RMSEA = 0.066, 95% CI: 0.018–0.108). Short FES-I scores were positively correlated with Katz ADL (Spearman’s ρ = 0.589) and FRAIL scores (ρ = 0.618) and negatively correlated with Berg Balance Scale scores (ρ = −0.800; all *p* < 0.001). Known-groups comparisons were generally consistent with the prespecified hypotheses. In the multivariable regression analysis, lower BBS scores, higher FRAIL scores, and greater fear of falling were significantly associated with higher Short FES-I scores (adjusted R^2^ = 0.710). **Conclusions**: The Turkish Short FES-I demonstrated high internal consistency and preliminary evidence supporting its construct validity. Although the modified single-factor model showed improved fit after two data-driven residual covariances were introduced, these structural findings require confirmation in an independent sample. Test–retest reliability, measurement error, and responsiveness also remain to be evaluated.

## 1. Introduction

Falls represent one of the major health challenges associated with ageing. Beyond the immediate risk of injury, falls may lead to functional decline, loss of independence, increased healthcare needs, and reduced quality of life among older adults [1,2]. The consequences extend beyond the individual, as fall-related injuries may also place a substantial burden on healthcare systems [1].

Fear of falling and concerns about falling are related but conceptually distinct constructs. Fear primarily reflects an emotional response to the possibility of falling, whereas falls efficacy concerns an individual’s perceived ability to perform everyday activities without falling. These concerns may influence activity participation and encourage avoidance of activities perceived as unsafe, potentially contributing to physical deconditioning and further functional decline [3]. Therefore, multidimensional instruments that assess concerns about falling during everyday activities may provide more clinically informative data than single-item questions. The Short Falls Efficacy Scale-International (Short FES-I) was developed to provide such an assessment while reducing the respondent burden associated with longer instruments [4,5].

Although fear of falling, falls efficacy, and concerns about falling are related constructs, they are not interchangeable. Although the official name of the instrument includes the term “Falls Efficacy,” the Short FES-I specifically assesses concerns about falling during everyday activities rather than falls-related self-efficacy or general fear of falling. Fear of falling primarily reflects an emotional response to the possibility of falling and may be assessed through a general self-reported question. Accordingly, the term “concerns about falling” is used throughout this manuscript when referring to the construct measured by the Short FES-I.

Although the 16-item FES-I has demonstrated strong psychometric properties, its length may increase respondent burden, particularly among older adults and in studies involving multiple assessment instruments. To provide a shorter alternative, Kempen et al. developed the seven-item Short Falls Efficacy Scale–International (Short FES-I) [5]. In its original validation study, the Short FES-I demonstrated high internal consistency, good test–retest reliability, and a strong association with the full-length FES-I. It also distinguished older adults according to characteristics such as age, sex, previous falls, and overall fear of falling [5]. Its shorter administration may offer practical advantages in clinical and community-based settings where assessment time and respondent burden are important considerations.

The seven-item Short FES-I was developed as a shorter alternative to the original 16-item FES-I to reduce respondent burden while retaining an assessment of concerns about falling across everyday activities. Its shorter administration may be particularly advantageous in older populations and time-constrained clinical or community-based assessments.

The Turkish version of the 16-item FES-I has previously demonstrated satisfactory psychometric properties among community-dwelling older adults, including evidence of reliability and associations with functional independence, mobility, and balance [6]. However, evidence regarding the Turkish adaptation of the seven-item Short FES-I remains unavailable. Evaluating the psychometric properties of this shorter version is therefore important, particularly because its reduced item burden may facilitate its use in clinical and community-based settings.

Accordingly, this study aimed to evaluate the structural validity, internal consistency, and construct validity of the Turkish Short FES-I through hypothesis testing, including convergent validity and known-groups validity, among community-dwelling adults aged 65 years and older.

## 2. Materials and Methods

### 2.1. Participants

This cross-sectional methodological study, involving the cross-cultural adaptation and psychometric validation of the Turkish Short FES-I, was conducted between August 2025 and May 2026 among community-dwelling adults aged 65 years and older attending 16 randomly selected Family Health Centers in Yunusemre and Şehzadeler, the two central districts of Manisa, Türkiye.

Data collection was conducted on two designated days per week at each participating center. On these days, individuals aged ≥65 years who attended the Family Health Centers, met the eligibility criteria, and agreed to participate were consecutively recruited until the target sample size was reached. For the purposes of this study, community-dwelling older adults were defined as individuals living in their own homes rather than residing in nursing homes or other institutional care settings.

The measurement model used in this study was relatively simple, consisting of a single latent factor represented by seven items with four-category ordinal responses. A sample size of 200 participants was considered reasonable for evaluating this model using DWLS (Diagonally Weighted Least Squares) estimation in confirmatory factor analysis (CFA) with ordinal indicators [7,8]. A total of 200 individuals aged 65 years and older were included in the study. Face-to-face interviews and Berg Balance Scale (BBS) assessments were conducted by the same researchers, all of whom were physicians.

### 2.2. Inclusion and Exclusion Criteria

The inclusion criteria were being aged 65 years or older, living in the community, volunteering to participate in the study, and having sufficient cognitive and communicative ability to understand and respond to the study questions. No standardized cognitive screening instrument was administered. Cognitive eligibility was assessed by the researcher during the initial explanation of the study and the informed consent process. Participants were considered eligible if they were able to understand the information provided about the study, communicate appropriately with the researcher, and understand and respond appropriately to the study questions. Individuals who were unable to adequately understand or respond to the study questions were not included.

### 2.3. Instruments

The data collection instruments included a sociodemographic and health questionnaire, the Short FES-I, the Berg Balance Scale (BBS), the Katz Index of Independence in Activities of Daily Living (ADL), and the FRAIL Scale.

The sociodemographic and health questionnaire collected information on participants’ demographic characteristics, history of falls, fear of falling, exercise status, chronic diseases, and assistive-device use. Lifetime fall history was assessed by asking participants whether they had ever fallen, and the number of previous falls was also recorded. Fear of falling was assessed using the question, “How afraid are you of falling?” with four response categories: “not at all afraid,” “a little afraid,” “quite afraid,” and “very afraid.” Exercise status, chronic disease history, and assistive-device use were assessed through participant self-report.

Exercise status was assessed using a self-reported question asking whether participants performed at least 150 min of moderate-intensity aerobic physical activity per week (e.g., brisk walking, swimming, cycling, dancing, gardening, or household activities) or at least 75 min of vigorous-intensity aerobic physical activity per week. The response options were “Yes,” “No,” and “Sometimes.” For the known-groups analysis, participants responding “Yes” or “Sometimes” were combined into the “Exercises” category, whereas those responding “No” were classified as “Does not exercise.” Consequently, this variable represents self-reported exercise status rather than verified adherence to recommended physical activity levels.

#### 2.3.1. The Short Falls Efficacy Scale-International (Short FES-I)

The Short FES-I consists of seven items addressing concerns about falling during common daily and social activities. The items assess concerns about falling while getting dressed or undressed, taking a bath or shower, getting in or out of a chair, going up or down stairs, reaching for an object above head level or on the ground, walking up or down a slope, and attending a social event. Each item is rated on a four-point Likert scale ranging from 1 (not at all concerned) to 4 (very concerned). Item scores are summed to obtain a total score ranging from 7 to 28, with higher scores indicating greater concern about falling. Total scores are classified as low concern (7–8 points), moderate concern (9–13 points), and high concern (14–28 points) [5].

#### 2.3.2. The Berg Balance Scale (BBS)

The Berg Balance Scale (BBS) is a performance-based instrument designed to assess functional balance, particularly in older adults. It consists of 14 items assessing static and dynamic balance during functional tasks, including sitting, standing, transfers, reaching, turning, and standing on one leg. Each item is scored on a five-point ordinal scale ranging from 0 to 4, with 0 representing the lowest level of performance and 4 representing the highest level of performance. Item scores are summed to obtain a total score ranging from 0 to 56, with higher scores indicating better balance performance [9]. The Turkish version of the BBS has been demonstrated to be a valid and reliable instrument for assessing balance in older adults [10]. Based on the BBS total score, participants were classified as having a high (0–20 points), moderate (21–40 points), or low (41–56 points) risk of falling [11].

#### 2.3.3. Katz Index of Independence in Activities of Daily Living (ADL)

The Katz Index of Independence in Activities of Daily Living (Katz ADL) is a widely used instrument for assessing the level of independence in basic activities of daily living among older adults. It evaluates six basic activities: bathing, dressing, toileting, transferring, continence, and feeding. Each activity is scored as 0 if the individual is independent and 1 if dependent. The total score ranges from 0 to 6, with lower scores indicating greater functional independence. A total score of 0 indicates complete independence, scores of 1–3 indicate partial dependence, and scores of 4 or higher indicate severe dependence. The Turkish version of the six-item Katz ADL has been demonstrated to be a valid and reliable instrument for assessing functional independence in older adults [12].

#### 2.3.4. FRAIL Scale

The FRAIL Scale is a five-item screening instrument used to assess frailty in older adults. It evaluates five domains: Fatigue, Resistance, Ambulation, Illnesses, and Loss of weight. Each component is scored as 0 or 1, yielding a total score ranging from 0 to 5, with higher scores indicating greater frailty. Participants are classified as robust (0 points), pre-frail (1–2 points), or frail (3–5 points). The Turkish version of the FRAIL Scale has been demonstrated to be a valid and reliable screening instrument for assessing frailty in older adults [13].

## 3. Procedure

### 3.1. Translation and Adaptation of the Short FES-I into Turkish

Following permission from the original developers, two independent forward translations of the Short FES-I from English into Turkish were performed by two physicians whose native language was Turkish and who had graduated from English-medium medical schools. Both translators were proficient in Turkish and English. The research team reviewed the two translations and resolved discrepancies through discussion and consensus, focusing on conceptual equivalence, linguistic accuracy, and comprehensibility. This process resulted in a preliminary Turkish version.

The preliminary Turkish version was subsequently back-translated into English independently by two native English speakers who were proficient in both English and Turkish and were not healthcare professionals. The back-translators were blinded to the original English version of the Short FES-I and had not seen the original scale items before performing the back-translation. The research team reviewed the back-translations and reached a consensus regarding the accuracy of meaning and clarity of the Turkish wording. No separate expert committee was convened to formally evaluate semantic, idiomatic, experiential, and conceptual equivalence. Instead, the translation and reconciliation stages were reviewed by the research team through discussion and consensus.

The resulting Turkish version was submitted to the original developers of the Short FES-I, and their approval was obtained.

Following this process, individual cognitive debriefing interviews were conducted with five adults aged 65–75 years to assess the comprehensibility of the Turkish version. The participants included three women and two men. Regarding educational attainment, two participants were primary school graduates, one was a middle school graduate, one was a high school graduate, and one was a university graduate. During the interviews, participants were asked whether any items or expressions were difficult to understand. All five participants reported that the items were clear and understandable, and no comprehension difficulties were identified. Consequently, no wording modifications were made following the cognitive debriefing interviews. These interviews were intended to provide preliminary evidence of item comprehensibility rather than to establish comprehensive cultural equivalence. The final Turkish version of the scale is provided in Appendix A.

### 3.2. Psychometric Analyses

The psychometric evaluation was organized according to measurement-property terminology consistent with the COSMIN framework. Structural validity was examined using confirmatory factor analysis (CFA), and internal consistency was evaluated using Cronbach’s alpha and McDonald’s omega coefficients. Construct validity was assessed through hypothesis testing, including correlations with related measures and known-groups comparisons. Test–retest reliability, measurement error, and responsiveness were not evaluated in this study.

#### 3.2.1. Distribution Characteristics

Floor and ceiling effects were evaluated by calculating the proportions of participants obtaining the minimum and maximum possible Short FES-I scores, respectively. Following the methodological criteria proposed by Terwee et al., a floor or ceiling effect was considered present when more than 15% of participants obtained the lowest or highest possible score, respectively [14]. Skewness and kurtosis coefficients were examined descriptively to characterize the distribution of Short FES-I scores, without applying a strict cutoff to determine normality.

#### 3.2.2. Reliability Analysis

Internal consistency was evaluated using Cronbach’s alpha and McDonald’s omega. Corrected item–total correlations and Cronbach’s alpha if item deleted were also examined.

#### 3.2.3. Validity Analyses

The validity of the Short FES-I was evaluated in terms of convergent validity, structural validity, and known-groups validity.

i. Convergent validity

Normality of the Short FES-I total score was assessed using the Kolmogorov–Smirnov test, which indicated a statistically significant deviation from normality (D = 0.125, *p* < 0.001). Although skewness (0.609) and kurtosis (−0.344) were within commonly accepted ranges, the bounded and discrete nature of the Short FES-I total score, together with the normality test results, supported the use of rank-based nonparametric methods for correlation and known-groups analyses.

Convergent validity was assessed by examining the relationships between the Short FES-I total score and the total scores of the Katz Index of Independence in Activities of Daily Living (KATZ), FRAIL Scale, and Berg Balance Scale. Since the scale scores did not meet the assumptions for parametric correlation analysis, Spearman’s rank correlation coefficient (Spearman’s rho) was used to evaluate these relationships.

A priori hypotheses were formulated regarding the expected direction of the associations. Higher Short FES-I scores were hypothesized to be associated with lower BBS scores, indicating poorer balance, and with higher FRAIL and Katz ADL scores, indicating greater frailty and functional dependency, respectively. Specific hypotheses regarding the magnitude of these correlations were not predefined.

These hypotheses were formulated before the analyses and concerned the expected direction of the associations. No numerical thresholds for the expected correlation magnitudes or minimum criteria for hypothesis confirmation were prespecified. Therefore, hypothesis testing was evaluated in relation to the predefined directions of association, while the observed correlation magnitudes were interpreted descriptively.

ii. Structural validity

Confirmatory factor analysis (CFA)

Descriptive statistics, internal consistency analyses, correlation analyses, group comparisons, and multiple linear regression analyses were performed using IBM SPSS Statistics (version 23.0; IBM Corp., Armonk, NY, USA). Confirmatory factor analysis was conducted separately using jamovi, as described below.

Confirmatory factor analysis (CFA) was performed using jamovi (version 2.6.26; The jamovi project, Sydney, Australia)), with the SEMLj module interfacing with the lavaan package in R. The seven Short FES-I items were treated as ordered categorical variables, and the single-factor model was estimated using diagonally weighted least squares (DWLS). The analysis used listwise deletion for missing observations, standard errors, and an unscaled model chi-square test, as specified in the SEMLj estimation settings. Model fit was evaluated using the chi-square statistic, degrees of freedom, comparative fit index (CFI), Tucker–Lewis index (TLI), normed fit index (NFI), standardized root mean square residual (SRMR), and root mean square error of approximation (RMSEA) with its 95% confidence interval. The initially specified model was evaluated without correlated residuals. Subsequently, two post-hoc residual covariances were introduced between Items 4 and 6 and between Items 5 and 7 based on modification indices. Both models were estimated using the same dataset and estimation settings.

iii. Known-groups validity

Known-groups validity was evaluated based on eight a priori hypotheses concerning differences in Short FES-I total scores across groups defined by age, sex, fall history, exercise status, fear of falling, functional dependency, balance-related fall risk, and frailty. Specifically, we hypothesized that Short FES-I scores would be higher among (1) older age groups, (2) women compared with men, (3) participants with a greater number of previous falls, (4) participants who did not exercise compared with those who exercised, (5) participants reporting greater fear of falling, (6) participants with greater functional dependency according to the Katz ADL, (7) participants classified as having higher fall risk according to the Berg Balance Scale (BBS), and (8) participants with greater frailty according to the FRAIL scale.

For these comparisons, age was categorized as 65–74, 75–84, and ≥85 years; fall history as no falls, one fall, and two or more falls; and fear of falling as not at all afraid, a little afraid, quite afraid, and very afraid. Sex was categorized as female or male, and exercise status as exercising or not exercising. Functional dependency was classified as fully independent, partially dependent, or highly dependent according to the Katz ADL; fall risk as low, moderate, or high according to the BBS; and frailty as robust, pre-frail, or frail according to the FRAIL scale. The Mann–Whitney U test was used for comparisons between two independent groups, whereas the Kruskal–Wallis test was used for comparisons involving three or more independent groups. Significant Kruskal–Wallis results were followed by pairwise Mann–Whitney U tests with Bonferroni correction for multiple comparisons. The adjusted significance threshold was set at *p* < 0.0167 for comparisons involving three groups (three pairwise tests) and *p* < 0.0083 for comparisons involving four groups (six pairwise tests).

Effect sizes were calculated for the known-groups comparisons. Rank-biserial correlations were used for Mann–Whitney U tests, and epsilon-squared (ε^2^) effect sizes were calculated for Kruskal–Wallis tests. Stratified percentile bootstrap 95% confidence intervals were estimated using 2500 resamples, preserving the original group sizes.

#### 3.2.4. Factors Associated with Short FES-I Scores

Multiple linear regression analysis was performed as a supplementary analysis to examine factors associated with Short FES-I total scores after mutual adjustment. The Short FES-I total score was entered as the dependent variable. Four variables were selected a priori on conceptual grounds: Berg Balance Scale (BBS) total score, FRAIL total score, Katz ADL total score, and fear of falling. Fear of falling was treated as a categorical variable with four response categories (“not at all afraid,” “a little afraid,” “quite afraid,” and “very afraid”). The “not at all afraid” category was used as the reference group, and three indicator variables were included in the regression model.

Model diagnostics included assessments of model specification, heteroskedasticity, residual normality, multicollinearity, and potentially influential observations. Multicollinearity was evaluated using variance inflation factors (VIFs), with values below 5 considered acceptable, and potentially influential observations were identified using Cook’s distance, with 4/n as the screening threshold. Given the evidence of heteroskedasticity, heteroskedasticity-consistent (HC3) covariance estimation was used for coefficient inference and the overall robust Wald F test. A sensitivity analysis was performed by refitting the model after excluding observations exceeding the Cook’s distance threshold. The sensitivity analysis was used to assess the stability of the associations, while the full sample was retained for the primary analysis.

Unstandardized regression coefficients (B), HC3 robust standard errors, 95% confidence intervals, and *p*-values were reported. Model explanatory performance was summarized using R^2^ and adjusted R^2^. Because the study was cross-sectional, regression coefficients were interpreted as adjusted associations rather than causal or prospective predictive effects. Individual Family Health Center identifiers were not available in the dataset; therefore, potential within-center clustering could not be evaluated or accounted for in the standard errors.

### 3.3. Ethical Issues and Permissions

All participants provided informed consent to participate in the study. Ethical approval was obtained from the Health Sciences Ethics Committee of Manisa Celal Bayar University (approval number: 20.478.486/3278, dated 9 July 2025).

## 4. Results

The mean age of the participants was 76.00 ± 8.56 years, and 56.5% were female. Most participants were retired (86.0%), 65.0% were married, and 47.5% reported that their income was equal to their expenses. At least one chronic disease was present in 90.0% of the participants; 70.5% used at least one assistive device, and 59.0% reported a history of falling (30.0% had experienced one fall, while 29.0% had experienced two or more falls). Of those who had experienced a fall, 28.0% had fallen within the past 6 months, while 50.8% had fallen within the past year. The median number of falls among those reporting falls was 1.0 (range: 1.0–20.0) (Table 1).

The distribution of the Short FES-I total scores showed slight positive skewness (skewness = 0.609) and a relatively platykurtic distribution (kurtosis = −0.344), with both values within acceptable limits. No substantial floor or ceiling effects were observed, as 10.5% of participants achieved the minimum score and only 0.5% achieved the maximum score. The mean Short FES-I total score was 13.38 ± 4.89, and the median was 13 (interquartile range [IQR]: 9–17). The observed scores ranged from 7 to 28. According to the predefined score thresholds, 36 participants (18.0%) were classified as having low concerns about falling (scores 7–8), 74 (37.0%) as having moderate concerns (scores 9–13), and 90 (45.0%) as having high concerns (scores 14–28).

The Short FES-I demonstrated high internal consistency, with a Cronbach’s alpha of 0.910 and a McDonald’s omega of 0.915. Corrected item–total correlations ranged from 0.608 to 0.839. Cronbach’s alpha if item deleted ranged from 0.884 to 0.909, indicating that removal of any individual item did not improve the internal consistency of the scale (Table 2).

Confirmatory factor analysis was conducted using the diagonally weighted least squares (DWLS) estimator to evaluate the proposed seven-item, single-factor structure of the Turkish Short FES-I. The initially specified model, without correlated residuals, yielded mixed fit indices: χ^2^(14) = 49.4, *p* < 0.001, CFI = 0.996, TLI = 0.994, NFI = 0.994, SRMR = 0.069, and RMSEA = 0.113 (95% CI: 0.080–0.148). Although the incremental fit indices were high, the RMSEA indicated inadequate fit according to commonly used criteria.

**Figure 1:**
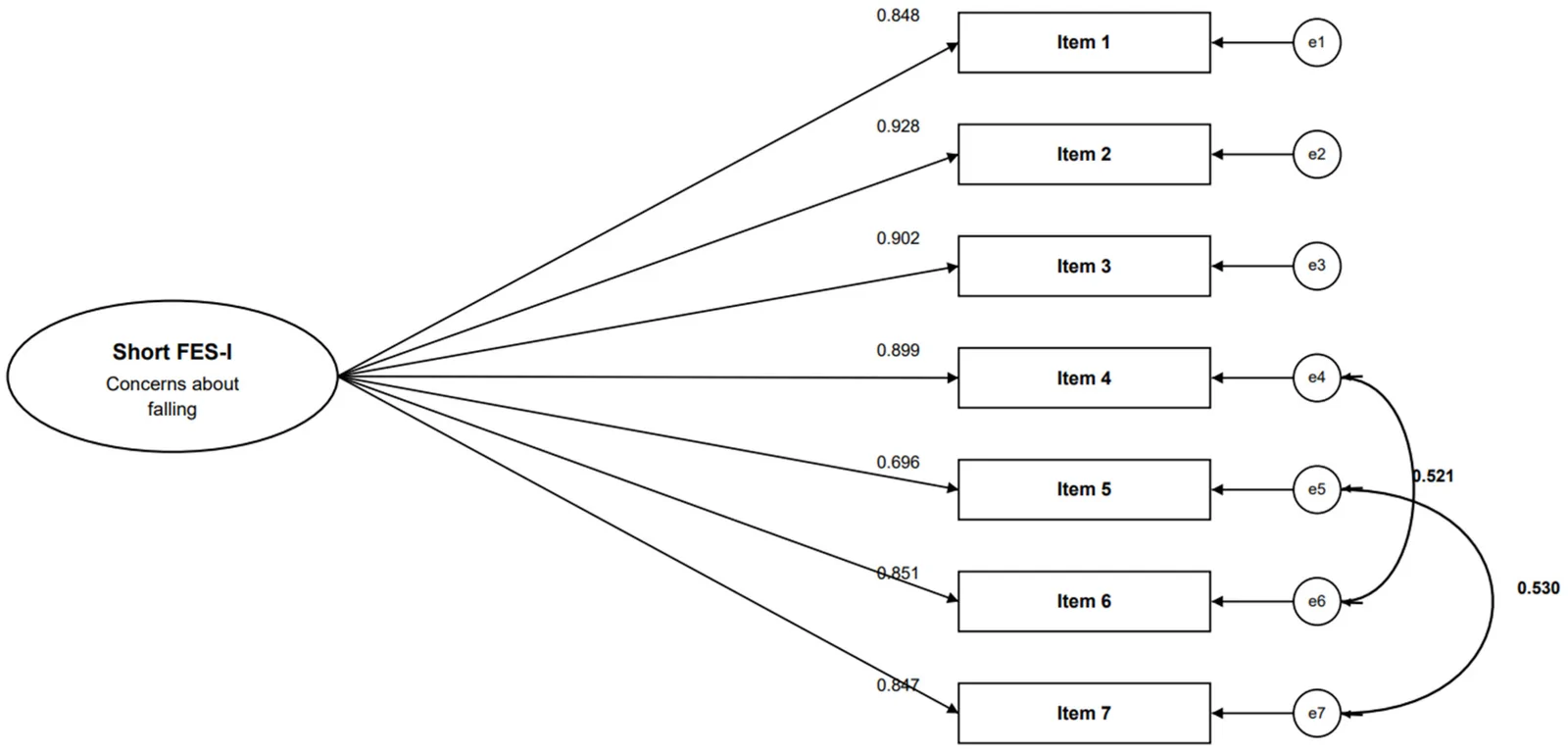
Modified single-factor confirmatory factor analysis model of the Turkish Short Falls Efficacy Scale–International (Short FES-I). Standardized factor loadings and standardized residual covariances are presented. Two post-hoc residual covariances were specified between Items 4 and 6 and between Items 5 and 7. The model was estimated using diagonally weighted least squares (DWLS; n = 200).

Inspection of the modification indices identified residual associations between Items 5 and 7 (MI = 17.847) and Items 4 and 6 (MI = 12.034). After introducing these two post-hoc correlated residuals, model fit improved: χ^2^(12) = 22.4, *p* = 0.033, CFI = 0.999, TLI = 0.998, NFI = 0.997, SRMR = 0.045, and RMSEA = 0.066 (95% CI: 0.018–0.108). Compared with the initial model, RMSEA decreased from 0.113 to 0.066 and SRMR from 0.069 to 0.045 (Table 3). Standardized factor loadings ranged from 0.749 to 0.941 in the initial model and from 0.696 to 0.928 in the modified model. Although these findings indicate improved fit for the modified single-factor model, the modifications were data-driven and require independent validation. The modified single-factor CFA model, including the two correlated residual pairs, is illustrated in Figure 1.

Although the modified model showed improved fit indices, the RMSEA estimate of 0.066 had a relatively wide 95% confidence interval (0.018–0.108), with an upper bound exceeding commonly used thresholds for acceptable fit. Therefore, uncertainty regarding the degree of model fit remains, despite the favorable incremental fit indices and SRMR.

The Short FES-I total score was positively correlated with Katz ADL scores (Spearman’s ρ = 0.589, *p* < 0.001) and FRAIL scores (ρ = 0.618, *p* < 0.001), whereas a negative correlation was observed with Berg Balance Scale scores (ρ = −0.800, *p* < 0.001) (Table 4). All three observed correlations were consistent with the prespecified directional hypotheses. However, because no a priori thresholds for correlation magnitude were established, the findings cannot be interpreted as confirmation of hypotheses regarding the strength of these associations.

The multiple linear regression model was statistically significant based on the HC3 robust Wald F test (F(6,193) = 102.902, *p* < 0.001), explaining 71.9% of the variance in Short FES-I total scores (R^2^ = 0.719; adjusted R^2^ = 0.710). The conventional OLS F statistic was 82.196 (*p* < 0.001).

Higher FRAIL scores (B = 0.783, *p* < 0.001) and lower Berg Balance Scale scores (B = −0.166, *p* < 0.001) were significantly associated with higher short FES-I scores. Compared with participants reporting no fear of falling, those reporting a little fear (B = 1.014, *p* = 0.024), quite a lot of fear (B = 3.071, *p* < 0.001), or very high fear (B = 3.795, *p* < 0.001) had significantly higher adjusted Short FES-I scores. The Katz ADL total score was not significantly associated with Short FES-I scores (*p* = 0.892) (Table 5).

Regression diagnostics indicated evidence of heteroskedasticity according to the Breusch–Pagan test (*p* = 0.002), although the White test was not statistically significant (*p* = 0.150). The Shapiro–Wilk test indicated a deviation from residual normality (*p* = 0.013), whereas the Ramsey RESET test did not indicate significant evidence of model misspecification (*p* = 0.474). VIF values ranged from 1.67 to 3.25, indicating no severe multicollinearity. The maximum Cook’s distance was 0.078, and 10 observations exceeded the prespecified screening threshold of 4/n.

In the sensitivity analysis excluding these 10 observations (n = 190), the adjusted associations of FRAIL scores, Berg Balance Scale scores, and all three fear-of-falling categories remained statistically significant, whereas Katz ADL remained nonsignificant. Although some coefficient magnitudes changed, the directions of the associations were consistent with those observed in the full sample. The full-sample HC3 model was retained as the primary analysis, and detailed results of the sensitivity analysis are presented in Supplementary Table S1.

Short FES-I total scores differed significantly according to fall history, sex, exercise status, fear of falling, Katz ADL dependency level, Berg Balance Scale fall-risk category, FRAIL category, and age group (all *p* < 0.001). Higher median Short FES-I scores were observed among participants with recurrent falls, women, those who reported not exercising, those with greater functional dependency, those at higher risk of falling, frail participants, and older age groups. In particular, Short FES-I scores increased progressively with greater fear of falling, with median scores of 8, 10, 14, and 18 among participants reporting no fear, a little fear, quite a lot of fear, and very high fear, respectively (Kruskal–Wallis H = 97.767, *p* < 0.001). All six pairwise comparisons between the four fear-of-falling categories remained statistically significant after Bonferroni correction (unadjusted *p* < 0.001 for each comparison; Bonferroni-adjusted significance threshold: *p* < 0.0083) (Table 6).

Effect-size analyses indicated differences of varying magnitudes across the prespecified known groups. Epsilon-squared values ranged from 0.144 for lifetime number of falls to 0.484 for fear of falling. Rank-biserial correlations were 0.412 for sex and 0.718 for exercise status. Detailed effect-size estimates and their bootstrap 95% confidence intervals are provided in Table 6.

## 5. Discussion

In this study, the structural validity, internal consistency, and construct validity of the Turkish version of the seven-item Short FES-I were evaluated in community-dwelling older adults. The findings provide preliminary evidence regarding the structural and construct validity of the Turkish Short FES-I, together with evidence of high internal consistency. However, the modified factor structure requires independent confirmation, and test–retest reliability, measurement error, and responsiveness remain to be evaluated [15]. Overall, the Turkish Short FES-I appears to be a brief and practical instrument for assessing concerns about falling among community-dwelling older adults, although further studies are needed to establish its temporal stability and responsiveness.

The Turkish Short FES-I demonstrated high internal consistency, with a Cronbach’s alpha of 0.910 and a McDonald’s omega of 0.915. The consistency between these coefficients provides further evidence of strong internal consistency. The Cronbach’s alpha coefficient obtained in our sample was close to the value reported for the original instrument and was also consistent with findings from Taiwanese, Japanese, and Brazilian adaptations [5,16,17,18]. Furthermore, the corrected item–total correlation coefficients ranged from 0.608 to 0.839, and the removal of any individual item did not increase the overall Cronbach’s alpha coefficient. These findings suggest that all items contribute meaningfully to the construct of concerns about falling and demonstrate strong item–total associations. They are consistent with the item loadings reported in the Brazilian study (0.58–0.86) and the item homogeneity reported in the original FES-I studies [5,17]. In a study evaluating the scale in three languages within a multi-ethnic population, Cronbach’s alpha coefficients were 0.896 for the overall scale, 0.905 for the English version, 0.905 for the Mandarin Chinese version, and 0.794 for the Bahasa Melayu version [19]. Collectively, these findings support the high internal consistency of the Short FES-I across different cultural and linguistic settings.

Confirmatory factor analysis (CFA) was conducted to examine the fit of the original one-factor structure of the scale in the Turkish sample. Although the initial model demonstrated favorable incremental fit indices, its RMSEA indicated inadequate fit. Following the introduction of two post-hoc correlated residuals, model fit improved, with CFI = 0.999, TLI = 0.998, NFI = 0.997, SRMR = 0.045, RMSEA = 0.066 (95% CI: 0.018–0.108), and χ^2^/df = 1.87 [20]. These findings are broadly consistent with previous validation studies. In the Brazilian adaptation study, CFA results supported the one-factor structure of the Short FES-I, suggesting its suitability as a unidimensional instrument for assessing concerns about falling [17]. Similarly, Tan et al. reported evidence supporting the unidimensional structure across different language versions of the scale, with adequate factor loadings for all items [19]. Nevertheless, the improved model fit in the present study was achieved only after introducing two post-hoc correlated residuals identified and evaluated using the same sample. Furthermore, the relatively wide 95% confidence interval for RMSEA (0.018–0.108) indicates uncertainty regarding the degree of model fit. Therefore, these findings provide preliminary support for a predominantly unidimensional structure rather than definitive confirmation of the original unmodified model. Independent replication and cross-validation of the modified factor structure are warranted. The two post-hoc residual covariances may have some conceptual plausibility based on item content. Items 4 and 6 both involve negotiating changes in elevation, such as stairs and slopes, and may share balance-related demands beyond the general latent construct of concerns about falling. The residual association between Items 5 and 7 is less straightforward. Reaching for objects and attending social events may both involve context-dependent environmental and postural demands, although this interpretation remains speculative. Importantly, these explanations were considered retrospectively and do not constitute independent theoretical validation of the modifications. Because both residual covariances were identified using modification indices in the same sample, the improved model fit should be interpreted as preliminary and requires confirmation in an independent sample.

A strong negative correlation was observed between Short FES-I and Berg Balance Scale (BBS) scores (Spearman’s ρ = −0.800), supporting convergent validity while indicating substantial overlap between self-reported concerns about falling and performance-based balance in this sample. Similarly, Kamide et al. (2018) identified longer Timed Up and Go (TUG) times, an objective indicator of mobility and balance, as a factor associated with higher Short FES-I scores in a Japanese population [16]. In the present study, BBS scores remained significantly associated with Short FES-I scores after adjustment for frailty, functional dependency, and fear of falling. However, the magnitude of the correlation warrants cautious interpretation. Although the Short FES-I and BBS assess conceptually distinct constructs, their strong association suggests considerable shared variance. This relationship may partly reflect the characteristics of our sample, which was recruited through Family Health Centers and had a relatively high prevalence of chronic conditions and fall history. Accordingly, the observed association may not generalize to all community-dwelling older populations. Furthermore, the functional measures included in the regression model were interrelated: BBS correlated with FRAIL (ρ = −0.635) and Katz ADL (ρ = −0.686), while FRAIL correlated with Katz ADL (ρ = 0.571). Although multicollinearity diagnostics did not indicate severe multicollinearity, these correlations suggest that the measures capture overlapping dimensions of physical and functional status. Their adjusted associations should therefore be interpreted in the context of their simultaneous inclusion in the regression model rather than as evidence of distinct causal or prospective predictive effects.

The strong positive correlation between Short FES-I and FRAIL Scale scores, together with the significant adjusted association between frailty and Short FES-I scores, is consistent with previous findings. In the Taiwanese study, the seven-item Short FES-I was independently associated with physical frailty criteria, including weight loss, exhaustion, slow walking speed, and reduced grip strength, in logistic regression analyses [18]. These findings suggest that greater frailty is associated with greater concerns about falling.

Short FES-I total scores were positively associated with Katz ADL dependency scores in the bivariate analysis (Spearman’s ρ = 0.589); however, this association was no longer statistically significant after adjustment for balance, frailty, and fear of falling in the multivariable regression model. One possible explanation is the conceptual and statistical overlap among functional dependency, balance performance, and frailty, as reflected in the correlations described above. Although the variance inflation factors did not indicate severe multicollinearity, these measures capture related aspects of physical and functional status. Consequently, the regression coefficients represent conditional associations after accounting for the other variables included in the model rather than the overall relationships of each construct with concerns about falling. The nonsignificant adjusted association for Katz ADL should therefore be interpreted cautiously and should not be taken as evidence that functional dependency is unrelated to concerns about falling or that shared variance fully explains the finding.

As hypothesized, participants reporting two or more lifetime falls had significantly higher Short FES-I scores than those reporting a single fall or no lifetime falls. This finding is consistent with studies conducted in Japan and Taiwan, which also reported higher Short FES-I scores among older adults with a history of falls [16,18]. ROC analyses in these studies indicated greater discriminative ability of the Short FES-I for identifying individuals with multiple falls (AUC > 0.73) than those with a single fall. One possible explanation is that previous fall experiences may be associated with greater concerns about falling and avoidance of activities perceived as risky. However, the direction of this relationship cannot be established from our cross-sectional data. Individuals with greater concerns about falling may also modify or restrict their activities, potentially influencing their exposure to situations in which falls could occur.

Tan et al. reported that although concerns about falling were significantly greater among older adults with a history of falls in univariate analyses, this association was no longer statistically significant after adjustment for age and ethnicity in multivariable regression models [19]. The authors suggested that older adults with greater concerns about falling may restrict their activities as a protective strategy or receive caregiver or family support that limits exposure to potentially hazardous situations. These observations highlight the complex relationship between concerns about falling and actual fall events, which may also be influenced by behavioral adaptations and cultural or family support mechanisms.

Women had significantly higher Short FES-I scores than men, consistent with previous studies reporting greater concerns about falling among older women. Several explanations have been proposed, including differences in physical functioning, exposure to fall-related situations, and perceptions or reporting of health-related concerns [17]. However, these potential mechanisms were not directly examined in the present study. Therefore, the observed sex difference should be interpreted as an association rather than evidence of any specific biological, behavioral, or sociocultural mechanism.

Participants who reported exercising had significantly lower Short FES-I scores than those who did not exercise. This association is consistent with the possibility that physical activity may be related to better balance, muscle strength, and functional ability, potentially contributing to lower concerns about falling. However, reverse causality is also plausible: older adults with greater concerns about falling may be less likely to engage in physical activity because they perceive certain activities as unsafe. Furthermore, unmeasured factors, such as overall health status and mobility limitations, may influence both exercise participation and concerns about falling. Given the cross-sectional design, the present findings cannot establish the direction of this relationship. Longitudinal and intervention studies are needed to clarify whether physical activity influences concerns about falling.

Median Short FES-I scores increased significantly across older age groups, consistent with previous research, including the findings of Tan et al. [19]. This association may be related to age-associated differences in balance, muscle strength, mobility, and functional independence. However, these explanations should be considered cautiously because the present cross-sectional study cannot determine whether age-related physiological changes account for the observed differences. Other factors associated with advancing age, including comorbidity and previous fall experiences, may also contribute to this relationship.

An important practical contribution of the present study is the availability of a shorter Turkish-language instrument for assessing concerns about falling among community-dwelling older adults. Compared with the previously validated 16-item Turkish FES-I, the seven-item Short FES-I requires fewer responses and may therefore reduce respondent and administrative burden. This potential advantage may be particularly relevant in primary care settings, such as Family Health Centers, where time for comprehensive geriatric assessment may be limited. The shorter instrument may also be useful in community-based screening programs, home visits, and population-health surveys involving large numbers of older adults, especially when concerns about falling are assessed alongside multiple other health indicators. However, administration time, respondent burden, and direct agreement with the Turkish 16-item FES-I were not evaluated in the present study. Therefore, the practical advantages of the shorter version should be regarded as potential benefits requiring confirmation in future comparative studies.

## 6. Limitations of the Study

This study has several limitations. First, reliability assessment was limited to internal consistency, as test–retest reliability, measurement error, and responsiveness were not evaluated.

Second, participants were recruited from older adults attending Family Health Centers on designated data collection days. Although the centers were randomly selected from two central districts of Manisa, participants were not randomly sampled from the general older population. Therefore, individuals who more frequently utilized primary healthcare services may have been more likely to participate, potentially contributing to the relatively high prevalence of chronic conditions and fall history in the study sample.

Third, acceptable model fit in the confirmatory factor analysis was achieved only after introducing two post-hoc correlated residuals. Because these modifications were data-driven and were not cross-validated in an independent sample, the possibility of sample-specific fit or overfitting cannot be excluded. Furthermore, multiple psychometric and association analyses were conducted using the same sample of 200 participants without independent validation data. Therefore, the modified factor structure and related findings require replication in larger, independent samples.

Finally, the cross-sectional design precludes determination of the temporal direction of associations between Short FES-I scores and participant characteristics. In particular, the relationship between exercise participation and concerns about falling may be bidirectional, and residual confounding cannot be excluded. Therefore, the observed associations should be interpreted carefully.

## 7. Conclusions

The findings provide preliminary evidence supporting the internal consistency and construct validity, including structural and known-groups validity, of the Turkish Short FES-I among community-dwelling older adults recruited from Family Health Centers. The seven-item Turkish Short FES-I may therefore be a useful instrument for assessing concerns about falling in similar primary care populations.

As participants were recruited from Family Health Centers rather than randomly selected from the general older adult population, the applicability of these findings to other settings and older adult groups should be interpreted with caution. In addition, test–retest reliability, measurement error, and responsiveness were not evaluated. In particular, the modified single-factor structure should be regarded as preliminary and requires validation in an independent sample before definitive conclusions regarding structural validity can be drawn. Further research assessing these additional psychometric properties and comparing the Turkish Short FES-I with the Turkish 16-item FES-I would complement the evidence provided by the present study.

## Figures and Tables

**Table 1:** Sociodemographic characteristics of the study group (n = 200).

Variables		n	%
Age	Mean ± SD	76.00 ± 8.56	
Sex	Female	113	56.5
	Male	87	43.5
Education level	Illiterate	18	9.0
	Literate	30	15.0
	Primary school graduate	58	29.0
	Middle school graduate	24	12.0
	High school graduate	42	21.0
	University graduate	28	14.0
Employment status	Retired	172	86.0
	Employed and retired	6	3.0
	Employed, not retired	2	1.0
	Not employed, not retired	20	10.0
Marital status	Married	130	65.0
	Divorced	4	2.0
	Widowed	66	33.0
Perceived income status	Income much higher than expenses	10	5.0
	Income higher than expenses	48	24.0
	Income equal to expenses	95	47.5
	Income lower than expenses	37	18.5
	Income much lower than expenses	10	5.0
Living arrangement	Living alone	36	18.0
	With spouse only	110	55.0
	With children only	27	13.5
	With spouse and children	18	9.0
	Other	9	4.5
Presence of chronic disease	Yes	180	90.0
	No	20	10.0
Orthopedic condition	Yes	86	43.0
	No	114	57.0
Use of assistive device	Yes	141	70.5
	No	59	29.5
Assistive device used *			
Visual and hearing aids	Glasses/contact lenses	112	56.0
	Hearing aid	21	10.5
Mobility assistive devices	Cane	38	19.0
	Wheelchair	5	2.5
	Walker	9	4.5
Other orthopedic devices	Hip/knee prosthesis	28	14.0
Other devices		3	1.5
Lifetime fall history	Yes	118	59.0
	No	82	41.0
Most recent fall within the past 6 months (n = 118)	Yes	33	28.0
	No	85	72.0
Most recent fall within the past 12 months (n = 118)	Yes	60	50.8
	No	58	49.2
Lifetime number of falls among participants with a fall history	Median (min–max)	1.0 (1.0–20.0)	

* Multiple responses were allowed because participants could report more than one type of device. Percentages were calculated using the total study sample (n = 200) as the denominator; therefore, percentages across device types do not sum to 100%.

**Table 2:** Corrected item-total correlations and Cronbach’s alpha if item deleted for the Short FES-I.

Items	Corrected Item-Total Correlation	Cronbach’s Alpha If Item Deleted
Item 1. How concerned are you about falling when getting dressed or undressed?	0.736	0.897
Item 2. How concerned are you about falling when taking a bath or shower?	0.800	0.892
Item 3. How concerned are you about falling when getting in or out of a chair?	0.751	0.897
Item 4. How concerned are you about falling when going up or down stairs?	0.839	0.884
Item 5. How concerned are you about falling when reaching for something above your head or on the ground?	0.608	0.909
Item 6. How concerned are you about falling when walking up or down a slope (e.g., ramp at a building entrance)?	0.768	0.894
Item 7. How concerned are you about falling when going out to a social event (e.g., place of worship, café, association, family gathering, or meeting friends)?	0.700	0.901
Overall Cronbach’s alpha	0.910	

**Table 3:** Model fit indices and standardized factor loadings for the initial and modified single-factor CFA models of the Turkish Short FES-I (n = 200).

**Parameter**	**Initial Model**		**Modified Model**	
**Model Fit Indices**				
χ^2^	49.4		22.4	
Degrees of freedom (df)	14		12	
*p*-value	<0.001		0.033	
χ^2^/df	3.53		1.87	
CFI	0.996		0.999	
TLI	0.994		0.998	
NFI	0.994		0.997	
SRMR	0.069		0.045	
RMSEA	0.113		0.066	
RMSEA (95% CI)	0.080–0.148		0.018–0.108	
**Item**	**Initial Model β**	**Modified Model β**	**Modified Model B**	**SE (B)**
Item 1	0.836	0.848	1.000	-
Item 2	0.898	0.928	1.094	0.0396
Item 3	0.880	0.902	1.064	0.0398
Item 4	0.941	0.899	1.060	0.0389
Item 5	0.749	0.696	0.821	0.0413
Item 6	0.906	0.851	1.004	0.0405
Item 7	0.882	0.847	0.999	0.0418

Abbreviations: CFA, confirmatory factor analysis; CFI, comparative fit index; TLI, Tucker–Lewis index; NFI, normed fit index; SRMR, standardized root mean square residual; RMSEA, root mean square error of approximation; CI, confidence interval; DWLS, diagonally weighted least squares. Both models were estimated using DWLS. The initial model specified a single latent factor without correlated residuals. The modified model included two post-hoc correlated residuals between Items 4 and 6 (MI = 12.034) and between Items 5 and 7 (MI = 17.847). Modification indices were obtained from the initial model. Standardized factor loadings are reported for both models. β, standardized factor loading; B, unstandardized factor loading; SE (B), standard error of the unstandardized factor loading. Item 1 was fixed at 1.000 in the unstandardized solution for model identification. Standard errors of standardized factor loadings were not available.

**Table 4:** Spearman’s rank correlation coefficients between scale scores.

Scales	Short FES-I Total Score	KATZ Total Score	FRAIL Total Score	Berg Balance Scale Total Score
Short FES-I total score	1.000			
KATZ total score	0.589 *	1.000		
FRAIL total score	0.618 *	0.571 *	1.000	
Berg Balance Scale total score	−0.800 *	−0.686 *	−0.635 *	1.000

All coefficients are Spearman’s rank correlation coefficients (ρ). * *p* < 0.01.

**Table 5:** Multiple linear regression analysis of factors associated with Short FES-I scores.

Variable	B	HC3 SE	95% CI for B	*p*
Constant	16.659	1.446	13.808–19.511	<0.001
Katz ADL total score	−0.035	0.254	−0.535–0.466	0.892
FRAIL total score	0.783	0.210	0.370–1.196	<0.001
Berg Balance Scale total score	−0.166	0.026	−0.217 to −0.116	<0.001
Fear of falling: A little afraid	1.014	0.445	0.137–1.891	0.024
Fear of falling: Quite afraid	3.071	0.524	2.038–4.105	<0.001
Fear of falling: Very afraid	3.795	0.718	2.380–5.210	<0.001

Dependent variable: Short FES-I total score. B: unstandardized regression coefficient; HC3 SE: heteroskedasticity-consistent standard error; CI: confidence interval. Reference category for fear of falling: Not at all afraid. R^2^ = 0.719; adjusted R^2^ = 0.710; HC3 robust Wald F(6,193) = 102.902; *p* < 0.001.

**Table 6:** Comparison of Short FES-I total scores for known-groups validity.

Variables	Categories	n	Short FES-I Total Median Score, (Q1–Q3)	*p* (Post-Hoc)	Effect Size (95% Bootstrap CI)
Age	65–74 years (a)	96	10.0 (8.0–14.0)	<0.001 * (a < b < c) **	0.274 (0.182–0.386)
	75–84 years (b)	71	14.0 (11.0–18.0)		
	85+ years (c)	33	17.0 (15.0–20.0)		
Gender	Female	113	15.0 (10.5–19.0)	<0.001 **	0.412 (0.268–0.551)
	Male	87	10.0 (8.0–14.0)		
Lifetime number of falls	No falls (a)	82	10.0 (8.75–14.0)	<0.001 * (a = b) < c **	0.144 (0.073–0.245)
	One fall (b)	60	13.0 (9.0–18.0)		
	Two or more falls (c)	58	16.0 (12.75–19.0)		
Exercise status	Exercises (Yes/sometimes)	93	10.0 (8.0–12.0)	<0.001 **	0.718 (0.607–0.812)
	Does not exercise (No)	107	16.0 (13.0–19.0)		
Fear of falling	Not at all afraid (a)	34	8.0 (7.0–10.25)	<0.001 * (a < b < c < d) **	0.484 (0.380–0.591)
	A little afraid (b)	49	10.0 (8.5–12.0)		
	Quite afraid (c)	54	14.0 (12–17.0)		
	Very afraid (d)	63	18.0 (14.0–21.0)		
KATZ ADL	Fully independent (a)	130	10.0 (8.0–14.0)	<0.001 * (a < b < c) **	0.347 (0.265–0.443)
	Partially dependent (b)	55	16.0 (12.0–19.0)		
	Highly dependent (c)	15	21.0 (19.0–23.0)		
Berg Balance Scale (BBS)	High fall risk (a)	26	20.5 (18.0–22.25)	<0.001 * (a > b > c) **	0.483 (0.412–0.561)
	Moderate fall risk (b)	49	16.0 (13.0–19.0)		
	Low fall risk (c)	125	10.0 (8.0–13.0)		
FRAIL Scale	Robust (a)	40	9.0 (7.25–11.0)	<0.001 * (a < b < c) **	0.336 (0.241–0.449)
	Pre-frail (b)	100	12.0 (9.0–15.0)		
	Frail (c)	60	18.0 (14.0–21.0)		

* Kruskal–Wallis test; ** Mann–Whitney U test. Following significant Kruskal–Wallis tests, pairwise Mann–Whitney U tests were performed with Bonferroni correction. Statistical significance was assessed at *p* < 0.0167 for three-group comparisons and *p* < 0.0083 for four-group comparisons. Different letters connected by < or > indicate statistically significant pairwise differences in the direction shown; an equals sign indicates a nonsignificant pairwise difference after correction. Effect sizes are reported as rank-biserial correlation (r_rβ_) for Mann–Whitney U tests and epsilon-squared (ε^2^) for Kruskal–Wallis tests. The 95% confidence intervals (CIs) were estimated using stratified percentile bootstrapping with 2500 resamples.

## Data Availability

The data supporting the findings of this study are available from the corresponding author upon reasonable request. The data are not publicly available due to privacy and confidentiality considerations.

## Supplementary Materials

The following supporting information can be downloaded at: https://www.mdpi.com/article/10.3390/healthcare14213694/s1, Supplementary Table S1: Sensitivity analysis of factors associated with Short FES-I total scores.

## Appendix A. Turkish Version of the Short Falls Efficacy Scale-International (Short FES-I)

EK 1. Uluslararası Düşme Etkinlik Ölçeği Kısa Formu.

Aşağıda, düşme olasılığı ile ilgili ne kadar endişelendiğinizi öğrenmeye yönelik bazı sorular yer almaktadır. Lütfen, bu etkinlikleri genellikle nasıl yaptığınızı düşünerek yanıtlayınız. Eğer şu anda bu etkinlikleri yapmıyorsanız (örneğin, alışverişinizi başkası yapıyorsa), bu etkinliği yapacak olsaydınız düşme ihtimalinden ne kadar endişeleneceğinizi düşünerek cevap veriniz.

Her bir etkinlik için, düşme ihtimalinin sizi ne kadar endişelendireceğini düşünerek en yakın olan seçeneği işaretleyiniz.
