# Supplementary material for: Cross-Cultural Adaptation and Psychometric Evaluation of the Turkish Short Falls Efficacy Scale–International (Short FES-I) for Assessing Concerns About Falling in Community-Dwelling Older Adults
Source: Healthcare (Basel). 2026 Nov 1;14(21):3694. doi: 10.3390/healthcare14213694 (PMC13762799; doi:10.3390/healthcare14213694)
Supplement: Supplementary file 1 [file healthcare-14-03694-s001.zip › Supplementary Table S1.pdf]

Supplementary Table S1. Sensitivity analysis of factors associated with Short FES-I total scores

Comparison of the primary multiple linear regression model (n = 200) with the sensitivity model excluding 10 observations with Cook's distance > 4/n (n = 190).

Variable	Primary B	Primary HC3 SE	Primary 95% CI	Primary p	Sensitivity B	Sensitivity HC3 SE	Sensitivity 95% CI	Sensitivity p
Intercept	16.659	1.446	13.808 to 19.511	<0.001	15.951	1.332	13.323 to 18.580	<0.001
Katz ADL total score	-0.035	0.254	-0.535 to 0.466	0.892	0.126	0.197	-0.262 to 0.515	0.522
FRAIL total score	0.783	0.210	0.370 to 1.196	<0.001	0.922	0.171	0.585 to 1.258	<0.001
Berg Balance Scale total score	-0.166	0.026	-0.217 to -0.116	<0.001	-0.153	0.023	-0.199 to -0.107	<0.001
Fear of falling: A little afraid	1.014	0.445	0.137 to 1.891	0.024	0.796	0.394	0.020 to 1.573	0.045
Fear of falling: Quite afraid	3.071	0.524	2.038 to 4.105	<0.001	3.036	0.495	2.059 to 4.013	<0.001
Fear of falling: Very afraid	3.795	0.717	2.380 to 5.210	<0.001	3.175	0.636	1.920 to 4.429	<0.001

Notes. B, unstandardized coefficient; HC3 SE, heteroskedasticity-consistent standard error; CI, confidence interval. The reference group for fear of falling is "Not at all afraid." Two-sided p values and 95% confidence intervals use t distributions with residual degrees of freedom (193 for the primary model; 183 for the sensitivity model). The full sample was retained for the primary analysis.

Primary model: $R^2 = 0.719$; adjusted $R^2 = 0.710$; HC3 robust Wald $F(6,193) = 102.902$, $p < 0.001$. Sensitivity model: $R^2 = 0.780$; adjusted $R^2 = 0.772$; HC3 robust Wald $F(6,183) = 139.884$, $p < 0.001$.
